# Marine heatwave temperatures enhance larval performance but are meditated by paternal thermal history and inter-individual differences in the purple sea urchin, *Strongylocentrotus purpuratus*

**DOI:** 10.3389/fphys.2023.1230590

**Published:** 2023-08-04

**Authors:** Terence S. Leach, Gretchen E. Hofmann

**Affiliations:** Department of Ecology, Evolution, and Marine Biology, University of California, Santa Barbara, Santa Barbara, CA, United States

**Keywords:** paternal effect, marine heatwave (MHW), marine invertebrate development, thermal tolerance, sea urchin (*Strongylocentrotus purpuratus*)

## Abstract

Marine heatwave (MHW) events, characterized by periods of anomalous temperatures, are an increasingly prevalent threat to coastal marine ecosystems. Given the seasonal phenology of MHWs, the full extent of their biological consequences may depend on how these thermal stress events align with an organism’s reproductive cycle. In organisms with more complex life cycles (e.g., many marine invertebrate species) the alignment of adult and larval environments may be an important factor determining offspring success, setting the stage for MHW events to influence reproduction and development *in situ*. Here, the influence of MHW-like temperatures on the early development of the California purple sea urchin, *Strongylocentrotus purpuratus*, were explored within the context of paternal thermal history. Based on temperature data collected during MHW events seen in Southern California from 2014–2020, adult urchins were acclimated to either MHW or non-MHW temperatures for 28 days before their sperm was used to produce embryos that were subsequently raised under varying thermal conditions. Once offspring reached an early larval stage, the impact of paternal and offspring environments were assessed on two aspects of offspring performance: larval size and thermal tolerance. Exposure to elevated temperatures during early development resulted in larger, more thermally tolerant larvae, with further influences of paternal identity and thermal history, respectively. The alignment of paternal and offspring exposure to MHW temperatures had additional positive benefits on larval thermal tolerance, but this tolerance significantly decreased when their thermal experience mismatched. As the highest recorded temperatures within past MHW events have occurred during the gametogenesis of many kelp forest benthic marine invertebrate species, such as the purple sea urchin, such parental mediated impacts may represent important drivers of future recruitment and population composition for these species.

## 1 Introduction

Anthropogenic emissions of carbon dioxide threaten marine ecosystems on multiple, varied timescales (IPCC, 2023). On top of more gradual changes in ocean temperature and acidification predicted in the next century, the increasing occurrence of acute, extreme climatic events in the present adds variability to an already dynamic system (Harris et al., 2018). Many coastal marine ecosystems, such as kelp forests and coral reefs, are currently threatened by persistent periods of anomalously high temperatures (Sen Gupta et al., 2020). The frequency and severity of such events—referred to as marine heatwaves (MHWs)—has necessitated clear definition to compare MHW events from across the world’s oceans (Hobday et al., 2016). Biologically, the consequences of MHWs are dramatic, triggering major disturbances to ecosystem functioning via: mass mortality within specific populations (Harvell et al., 2019; Seuront et al., 2019) and species range shifts (Sanford et al., 2019), which contribute to altered community structure and biodiversity (Wernberg et al., 2016; Rogers-Bennett and Catton, 2019; Michaud et al., 2022). Predictive models suggest that more severe and longer-lasting temperature events will be an increasingly consistent occurrence for many marine ecosystems, further justifying efforts to quantify organismal responses to the associated stressors (Frolicher et al., 2018; Oliver et al., 2018).

The response to elevated temperatures, and many other environmental variables, varies across the life history of marine taxa and thus must be incorporated in predictions about adaptive capacity (Przeslawski et al., 2015; Thurman et al., 2020). This is especially true for benthic marine invertebrates, many of which possess complex life history strategies that involve the exploitation of different habitats in early and larval development (pelagic) compared to that of adults. Early developmental stages in these taxa are particularly vulnerable to environmental stress and have received a lot of attention given their hypothesized role as representing the “weak link” in an organism’s response to climatic changes (Byrne, 2011; Pandori and Sorte, 2019). The temperature experienced during early developmental stages, specifically, exhibits a large influence on survival and development (Sewell and Young, 1999; O’Connor et al., 2007; Byrne et al., 2009). Despite targeted efforts to elucidate the thermal sensitivity of marine invertebrate early development, much remains unknown about the mechanisms that foster resilience to thermal challenges. Early evidence suggests that tolerance mechanisms may span across generations, termed intergenerational plasticity, opening an avenue in which the impact of extreme events can persist long after the event takes place (Uller, 2008; Yin et al., 2019; Palombo et al., 2023). This intergenerational plasticity may have positive or negative consequences and is increasingly complex given the potential for both paternal and maternal influence.

For decades, maternal investment into egg quantity and composition was thought to be one of the only mechanisms of parental influence over offspring success - outside of underlying genetics (Mousseau and Fox, 1998). Indeed, maternal effects play a pivotal role in offspring performance, but the investigation of other non-genetic inheritance mechanisms, such as epigenetics, provides a new avenue in which both parents might contribute to the success of the next-generation (Immler, 2018; Eirin-Lopez and Putnam, 2019). This evidence has especially bolstered research into paternal effects, defined here as the mechanisms, outside of genetic inheritance, driving paternal impact, on offspring phenotype (Crean and Bonduriansky, 2014). At the sperm-level, males demonstrate a wide range of gamete plasticity that is influenced by both abiotic (pH, temperature, salinity, pollution) and biotic (nutrition and sperm competition) factors (reviewed in Marshall, 2015; Eads et al., 2016; Munari et al., 2022). This plasticity may be an adaptive strategy that promotes fertilization under varying environmental conditions. The influence of sperm plasticity may even extend into offspring development. Recent studies in marine tubeworms show that male acclimation alone to salinity and temperature influence both offspring survival and physiological performance (Jensen et al., 2014; Guillaume et al., 2016). This side of gamete plasticity becomes more interesting when considering the potential for a paternal equivalent to anticipatory maternal effects, where mothers in predictable environments can use cues to alter the resources given to their eggs in order to increase offspring fitness within that environment (Marshall and Uller, 2007). Given the shorter timescales of sperm production in many organisms, paternal effects are an increasingly interesting research topic for predicting the biological consequences of extreme events like MHWs.

For this study, we used an ecologically important benthic invertebrate (Pearse, 2006), the purple sea urchin, *Strongylocentrotus purpuratus*, to explore how paternal acclimation to MHW temperatures influenced offspring performance. The reproductive and developmental biology of *S. purpuratus*—a broadcast spawner with planktonic larvae—provides a means to isolate the effect of paternal environment alone while also facilitating investigation of an ecologically relevant scenario in which adults and their offspring experience varying environmental conditions. In the Santa Barbara Channel (SBC), the timing of purple sea urchin gametogenesis (beginning in early fall) coincides with the seasonality of MHW events experienced in the area over the last decade (Reed et al., 2016; Chamorro et al., 2023). As such, purple sea urchin populations may be carrying the effects of such events during a crucial portion of their reproductive cycle. Ecologically, purple sea urchins are a linchpin of kelp forest ecosystems across the western coast of the United States (Ebert, 2010; Yorke et al., 2019). Population dynamics of *S. purpuratus* heavily influence ecosystem state, facilitating shifts from dense kelp forests to kelp-depleted urchin barrens through their persistent grazing of young kelp recruits (Dayton et al., 1992). Thus, the influence of MHW events on *S. purpuratus* populations may have overarching consequences for the kelp forest ecosystems they inhabit, giving efforts to quantify their response large-scale applicability (Reed et al., 2016; Rogers-Bennett and Catton, 2019; Okamoto et al., 2020).

In this study, we sought to explore the role of paternal effects in *S. purpuratus* within the context of MHW events. Published work focused on sperm performance using this experimental design showed that male acclimation to MHW temperatures decreased fertilization success (Leach et al., 2021). Here, the research goal aims to shed light into whether such paternal effects will further extend into early development. Given the shorter timescales of spermatogenesis for many species, we hypothesized that paternal thermal stress under even relatively brief timescales would persist into the next-generation. To test this hypothesis, male sea urchins were acclimated to temperature conditions experienced in the SBC during recent MHW events and non-MHW time periods. Individual males from each acclimation temperature were then spawned and their sperm used to create embryos that were separated into the same two temperature treatments mentioned above. At the echinopluteus larval stage, offspring performance under varying rearing and sire thermal conditions was quantified in two ways: 1) thermal tolerance to an acute temperature exposure and 2) larval size.

## 2 Materials and methods

### 2.1 Urchin collection and sex identification

Collection and sex determination of the purple sea urchin, *S. purpuratus*, were conducted as described in Leach et al. (2021). In brief, sea urchins were hand-collected via SCUBA from two kelp forest sites in the Santa Barbara Channel: Mohawk Reef (34.394°N, 119.730°W) and Arroyo Quemado Reef (34.468°N, 120.119°W). Collections occurred on December 13th and 18 December 2019, respectively, using California Scientific Collection permit SC-“9228” (to the Santa Barbara Coastal LTER project). Following collection, sea urchins were transported to the seawater facilities at UC Santa Barbara’s Marine Science Institute and maintained at 13°C. The experimental design necessitated the identification of male and female *S. purpuratus,* therefore each sea urchin was sexed by a physical induction of spawning shortly after collection. This method successfully identified both male (n = 31) and female (n = 43) sea urchins. Individuals of each sex were subsequently separated into different recovery tanks, allowing for the tracking of sex and collection location identities throughout the study. Sea urchins recovered for 2 weeks at 13°C while being fed kelp (*Macrocystis pyrifera*) *ad libitum.* Over this time, there was minimal disease and/or death observed across both male and female sea urchins (∼97% survivorship).

### 2.2 Adult acclimation

After the recovery period concluded, male urchins were acclimated for 28 days to one of two temperature treatments mimicking the conditions experienced in the Santa Barbara Channel either: 1) during a MHW event (20°C) or 2) during non-MHW conditions (14°C). The design and structure of how treatment conditions were maintained are detailed in Leach et al. (2021). For brevity, each treatment was represented by three acclimation tanks, kept within one water table where temperature was regulated using a Delta Star pump with a Nema 4x digital temperature controller (AquaLogic). Temperatures within each tank were tightly monitored, every 5 min, with HOBO temperature loggers (Onset Computer Corporation) over the month-long acclimation. Each tank was further subdivided to support different rounds (n = 3 rounds) of male sea urchin acclimation, allowing for increased replication. During each round, two male urchins were placed in a subdivided portion of each acclimation tank and maintained for 28 days with a weekly feeding of kelp. Rounds were staggered by 96 h to ensure that each acclimated male experienced exactly 28 days at their respective temperature treatment. Alternatively, female sea urchins were maintained at ambient conditions, ∼13°C, and fed kelp *ad libitum* in multiple tanks for the entirety of the male acclimation period.

### 2.3 Urchin spawning and crossing design

At the completion of each round’s acclimation, male and female urchins were induced to spawn using a combination of intercoelomic injections of 0.53M KCl and physical perturbation (Strathmann, 1987). Male and female urchins were randomly selected from their tanks, with males from different replicate acclimation tanks represented within each round. Each urchin was injected with 1–3 mL of 0.53M KCl, depending on size, via multiple injections in the tissue surrounding the mouth. Following injection, the sea urchin was shaken out of water for at least 1 min. Spawned sperm was collected dry and kept on ice while eggs were collected by inverting female urchins over beakers filled with filtered seawater (FSW). Gamete quantity and quality were observed under a light microscope. The compatibility of gametes for every combination of spawned male and female urchin was assessed by conducting test fertilizations. Only male and female urchins demonstrating healthy gametes (good sperm motility/mature, normal looking eggs and >90% fertilization success during test fertilizations) were used for the offspring rearing portion of the experiment.

**FIGURE 1:**
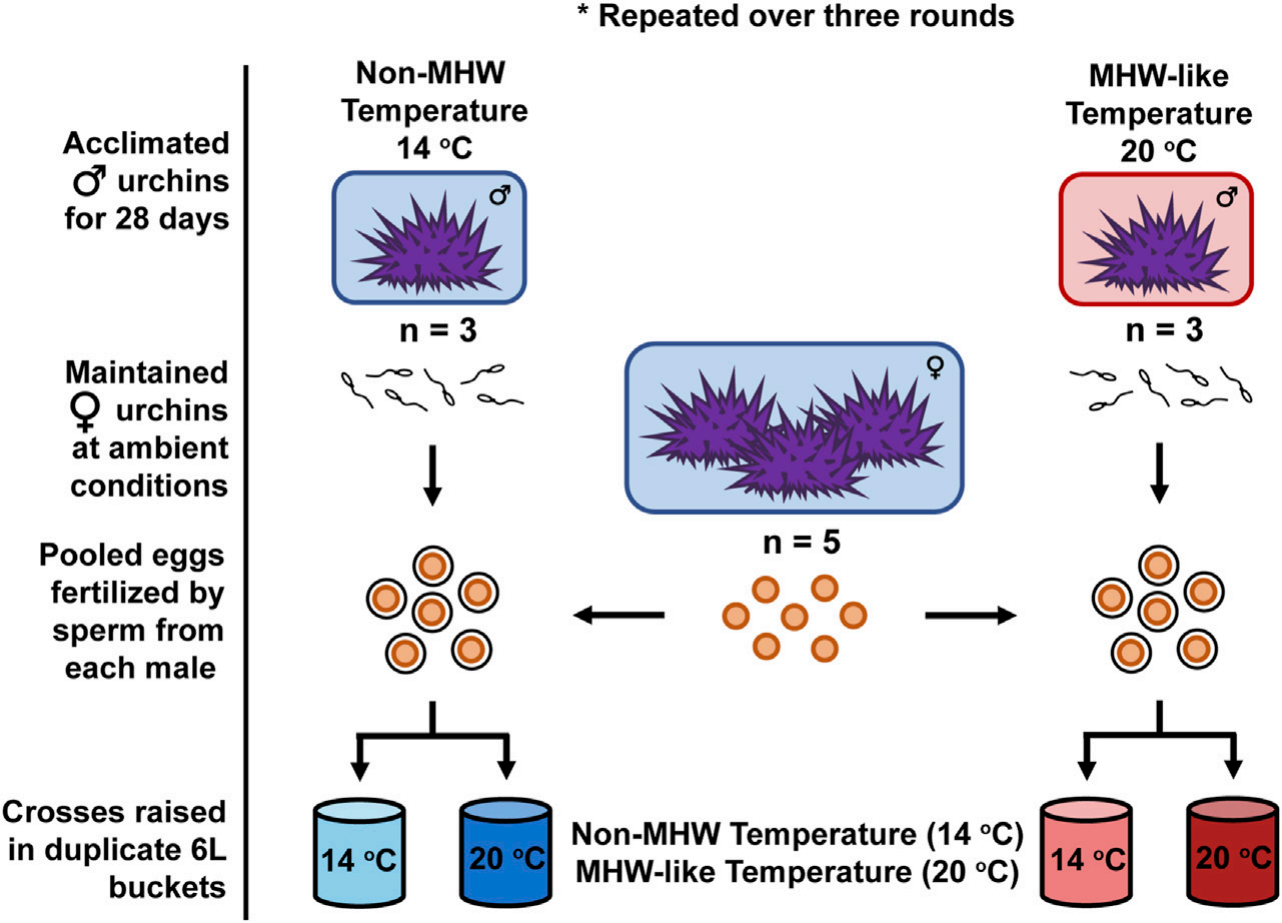
Experimental Design. Across three rounds, male purple sea urchins (*Strongylocentrotus purpuratus*) were acclimated to either a non-MHW (14°C) or MHW-like (20°C) temperature treatment for 28 days. In each round, sperm from individual males (n = 3 male urchins/temperature treatment) were collected and crossed with pooled eggs from five female urchins in a split-clutch breeding design. Fertilizations by each male and the resulting embryos were then raised under the same non-MHW (13°C) and MHW-like (20°C) temperature conditions experienced during adult acclimation. Offspring were raised under these conditions, in duplicate culture vessels, until being sampled at the echinopluteus larval stage for various performance metrics.

Gametes were crossed in a modified split-clutch design: involving individual fertilizations of pooled eggs, containing approximately equal numbers of eggs from each female urchin (n = 5 females), by sperm of three males from both the MHW-like and non-MHW temperature treatments. This resulted in 6 unique crosses (n = 3 males per treatment × 2 treatments), or families per round (Figure 1). The split-clutch design was utilized to minimize the effects of male × female interactions on fertilization and subsequent development. Across the three rounds, 9 males from each treatment and 3 pools of females were used for a total of 18 families. During each round, individual fertilizations by each male were conducted by titrating sperm into a 1L-beaker containing pooled eggs (∼300 eggs/mL) until >90% of the eggs were fertilized. Fertilizations for males acclimated to MHW-like or non-MHW temperatures were initiated 1 h apart from one another to ensure consistent sampling from the culture vessels (described below).

### 2.4 Larval culturing

Fertilized eggs produced by each cross (n = 3 crosses per paternal treatment per round) were subsequently used to stock two, flow-through culture vessels kept at either the same MHW-like or non-MHW temperature conditions used for the adult acclimation (Figure 1). This resulted in a total of 12 cultures (n = 6 cultures per larval rearing treatment) per round. Culture vessels consisted of two, nested 2.5-gallon buckets capable of holding 6 L total. The inner bucket, where developing urchins were held, contained eight, 5.5 cm holes covered with 64-micron mesh to permit water flow without the loss of embryos. The lid of each vessel served as a platform for a 12-V motor that powered an 8-cm by 10-cm acrylic paddle, which gently mixed cultures. Filtered, UV sterilized seawater (FSW) flowed into each culture vessel from a reservoir tank at a rate of 4 L/h via irrigation button drippers (DIG Corporation). Each culture vessel was half-way submerged in a large water bath where target temperatures were controlled using a Delta Star heat pump with a Nema ×4 digital temperature controller (AquaLogic). To maintain a favorable larval density of 10 embryos/mL (Strathmann, 1987), each 6-L culturing vessel was loaded with 60,000 embryos. Once loaded with sea urchin embryos, the temperature of each culture was monitored by regular point measurements using a wire thermocouple (Thermolyne PM 20700/Series 1218).

Cultures were monitored frequently to assess developmental progression. Sampling occurred once offspring reached an early echinopluteus stage, where larvae possessed a differentiated gut, skeletal rods, and the early formation of feeding arms (∼49 hpf at 20°C; ∼60 hpf at 13°C). Larvae were siphoned from culture vessels onto a submerged 35-micon mesh filter before being transferred, using a plastic transfer pipette, to a 15-mL Falcon tube. Larval concentrations within the Falcon tubes were estimated by counting the number of individuals within three small aliquots, where a coefficient of variance (CV) of <10% was reached. The average number of larvae across aliquots was used to represent the new concentration, or the number of larvae per mL of FSW. Subsamples were then collected for various downstream phenotypic quantifications based off these concentrations.

### 2.5 Thermal tolerance assay

The influence of paternal and offspring rearing temperature on echinopluteus thermal tolerance was assessed following a modified protocol established by Hammond and Hofmann (2010). Larvae from each sample were divided and exposed to one of 6 different temperatures for a short period of time and then assessments of survival and normality were conducted using microscopy. Within offspring temperature treatments, larvae from each individual male were pooled across duplicate culture vessels. 6,000 of these pooled larvae were then divided across six, 20-mL glass scintillation vials so that each contained 1,000 larvae in 5 mL of FSW (200 larvae/mL). These scintillation vials were placed in an aluminum block possessing a temperature gradient from 16–30°C. This temperature gradient was created by attaching two water baths set at a high and low temperature at either end of the aluminum block. Larvae within each scintillation vial were exposed to 5 different assay temperatures (27.0 ± 0.3°C, 27.9 ± 0.3°C, 28.9 ± 0.3°C, 29.9 ± 0.2°C, 31.2 ± 0.3°C), for 1 h before being removed from the temperature block. While these temperatures do not reflect ecologically relevant scenarios for *S. purpuratus* within the SBC, they were instead selected to explore the influence of paternal and offspring rearing temperature on the underlying thermal physiology of urchin larvae. 100 larvae from each vial were then coded and scored blind (each vial had a random number ID) for mortality.

Here, mortality was based upon two criteria 1) the lack of ciliary movement and 2) abnormality, more specifically the presence of ruptured membranes or enlarged cells obstructing the gut lining. As the degree of abnormality did not appear reversible, only healthy-looking larvae with moving cilia were scored as alive. Statistical analysis of thermal tolerance followed that outlined in Waite and Sorte (2022). A binomial regression was performed using binary mortality data (alive vs dead) across assay temperatures to calculate lethal thermal limits (LT_50_) of larvae, or the temperature in which 50% of larvae in each treatment died (de Vries et al., 2008; Bates et al., 2015). This method was used to additionally calculate LT_25_ and LT_10_ values to gain a broader sense of thermal sensitivity. A generalized mixed effects model with a Gamma distribution was then used to assess how larval thermal tolerance (LT_50_) was affected by offspring and paternal temperature. The model was produced using the lme4 package (Bates et al., 2015) and included paternal temperature, offspring rearing temperature, and their interaction as fixed factors, while male identity and round of the experiment were included as random factors.

### 2.6 Morphometric analysis

Larval size was measured as a trait that could be impacted by paternal and offspring rearing temperature. For the analysis, 500 larvae were sampled for each culture vessel and preserved using 4% formalin in 0.01 M phosphate buffered saline (PBS) pH 8.7. This solution was used to minimize dissolution of skeletal rods within echinopluteus larvae. The fixative and larvae were added in equal parts so that the final concentration of formalin was 2%. Preserved larvae were stored at 4°C prior to processing. Larvae were digitally photographed under a compound microscope using an attached digital camera (Infinity Lite) and Infinity Capture software (version 6.2.0). Using ImageJ (National Institutes of Health, United States), individual larvae from each culture vessel (n > 30) were measured. Only echinopluteus larvae positioned in the correct orientation, where the length of the postoral arm of the plutei, from the spicule tip of the postoral arm to the spicule tip of the aboral point, were photographed and measured (Yu et al., 2011).

Images were taken for all larvae in the above orientation, regardless of which postoral arm, left or right, was in focus. Later analysis showed that at this stage of early pluteus, there was no asymmetry in spicule length between left or right postoral arms, so all larvae imaged were included in analysis. Differences in arm length, spicule length, and the ratio of the spicule length to body length between treatments were compared using two-way ANOVAs. Here, the linear mixed effects model included paternal acclimation temperature, offspring rearing temperature, male identity, as well as the interactions of offspring rearing temperature with paternal temperature and male identity, separately, as fixed factors. Pooled eggs used for this experiment decreased in size (diameter and area) between each round, the cause remaining unclear. Average diameter decreased from 95.9 ± 4.8 µm in Round 1 to 93.3 ± 2.5 µm and 91.5 ± 3.1 µm in Rounds 2 and 3, respectively. As such, Round was accounted for as a random effect in all statistical analysis of the various morphological features. Statistical analyses were performed in R.

## 3 Results

### 3.1 Adult acclimation and larval culturing

Adult acclimation in the lab was successful and resulted in healthy, gravid animals. Specifically, no mortality was observed in either the high or low temperature treatment over the month-long acclimation period. In addition, temperature conditions remained relatively stable across these acclimations with a ∼6°C separation between MHW-like and non-MHW temperature treatments (Table 1). In addition, all adult male urchins released sperm when spawned in the laboratory, further indicating that the acclimation period and time in the lab supported gametogenesis.

**TABLE 1:** Temperatures recorded during adult acclimations and larval rearing. All values displayed as mean ± standard deviation.

Acclimation	Treatment	Duration	Temperature (^o^C)
Adult	MHW-like	28 days	19.6 ± 0.2
	non-MHW	28 days	13.5 ± 0.4
Development	MHW-like	48–60 h	19.5 ± 0.4
	non-MHW	48–60 h	13.3 ± 0.3

Other aspects of the overall experiment were also successful. During larval culturing, temperature conditions under which the embryos and larvae were reared closely matched the temperatures used for adult acclimation (Table 1). Additionally, the development in the culturing phase was synchronous across rearing temperature treatments; however, there was variation in the degrees of larval loss (either due to mortality or human error). There were no substantial differences in larval loss between paternal temperature treatments during any of the rounds. Overall, larvae from 70 out of 72 culture vessels were represented in all the down-stream analyses described below.

### 3.2 Thermal tolerance

**FIGURE 2:**
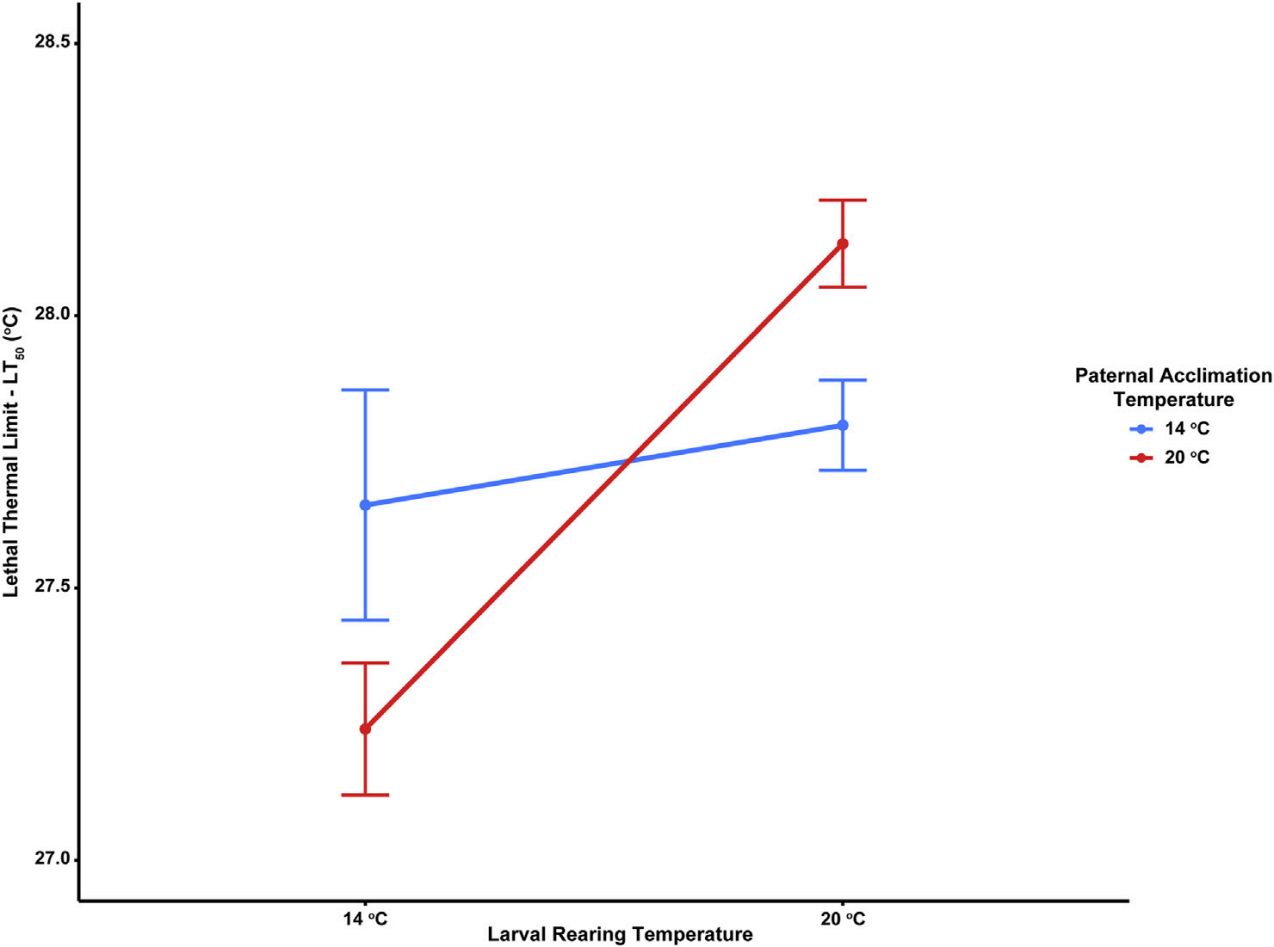
Effect of paternal acclimation (non-MHW, 14°C; MHW-like, 20°C) and larval rearing temperature (non-MHW, 13°C; MHW-like, 20°C) treatments on the lethal thermal limit (LT_50_) of echinopluteus larvae in the purple sea urchin, *Strongylocentrotus purpuratus*. Data points represent average LT_50_ across larvae from individual males (n = 9 male urchins/paternal treatment) and error bars represent mean ± standard error.

Thermal tolerance assays yielded interesting, but complex results, with LT_50_ values significantly influenced by the interacting effects of adult acclimation temperature and the temperature at which embryos developed. LT_50_ values across treatments ranged from 27.2–28.1°C (Table 2). There was a minimal effect of paternal temperature alone on larval thermal tolerance (Table 3; F_1,16_ = 0.006, *p* = 0.938). Despite this, LT_50_ values were significantly influenced by the interaction between paternal and larval temperatures (Table 3; F_1,16_ = 12.13, *p* = 0.002). Here, larval thermal tolerance was greatest when the thermal environments experienced by the sire and their offspring matched (Figure 2). Larvae from 20°C-acclimated sires exhibited a near 1°C increase in LT_50_ when reared under the 20°C treatment (28.1 ± 0.1°C) as compared to the 14°C treatment (27.2 ± 0.1°C). Alternatively, there was fairly little variation in the LT_50_ values of larvae produced by 14°C-acclimated sires when they developed under either rearing temperature (27.7 ± 0.2°C vs 27.8 ± 0.1°C). Additionally, developmental temperature alone significantly influenced larval LT_50_, with larvae raised under the MHW-like temperature treatment (20°C) possessing higher thermal tolerance than those raised under the non-MHW temperature treatment (14°C) (F_1,16_ = 12.59, *p* = 0.001; Figure 2). Finally, the trends outlined above regarding the match of sire and offspring thermal exposure with larval thermal tolerance were also observed in calculated LT_25_ and LT_10_ values (Table 2).

**TABLE 2:** LT_50_, LT_25_, and LT_10_ values for echinopluteus larvae from various paternal and larval treatments. All values given as mean ± standard deviation.

Temperature treatment (sire - offspring)	LT_10_	LT_25_	LT_50_
14°C–14°C	26.6 ± 0.3	27.1 ± 0.2	27.7 ± 0.2
14°C–20°C	26.9 ± 0.2	27.4 ± 0.1	27.8 ± 0.1
20°C–14°C	26.7 ± 0.3	27.0 ± 0.2	27.2 ± 0.1
20°C–20°C	27.3 ± 0.2	27.7 ± 0.1	28.1 ± 0.1

**TABLE 3:** Statistical output from generalized linear mixed-effects model analyzing the effect of paternal and larval temperature treatments on LT_50_ values of *Strongylocentrotus purpuratus* echinopluteus. Significant *p*-values are denoted in bold and with an asterisk.

	*F*	*df*	*p*
Paternal Temperature Treatment	0.006	1,16	0.938
Larval Temperature Treatment	12.591	1,16	0.001*
Paternal Temperature Treatment * Larval Temperature Treatment	12.135	1,16	0.002*

### 3.3 Morphometrics

**FIGURE 3:**
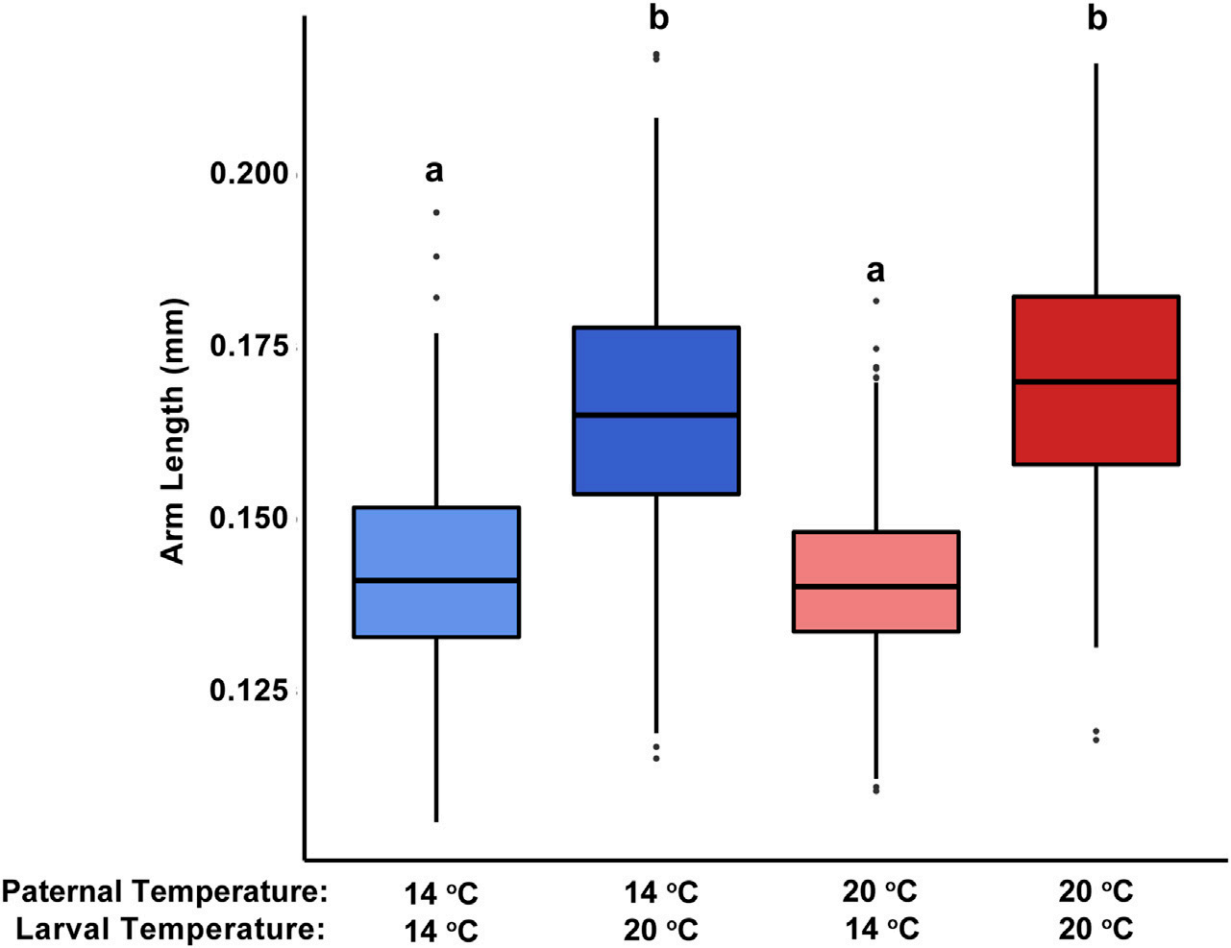
Effect of paternal and offspring temperature treatments on larval arm length in the purple sea urchin, *Strongylocentrotus purpuratus*. Bars show standard error of the mean. Different letters denote significant differences (*p* < 0.001) in arm length between treatments, based on a *post hoc* Tukey test.

In general, as factors that could alter morphometrics, developmental conditions had a greater influence on larval body form than did the thermal history of the sire. Pluteus arm length was significantly influenced by the temperature at which development occurred (F_1,1_ = 538.92, *p* < 0.001). Alternatively, paternal acclimation temperature (F_1,1_ = 1.65, *p* = 0.208) and the interaction between paternal and larval temperature treatments (F_1,1_ = 3.78, *p* = 0.104) had more minimal effects. Regardless of paternal acclimation, pluteus reared under 20°C possessed much longer arms (179.3 ± 1.6 and 182.2 ± 1.9 um for larvae from 20°C- to 14°C-acclimated sires, respectively) than those reared under 14°C (143.6 ± 3.4 um and 141.5 ± 2.1 um; Figure 3). These trends were consistent across the other metrics used to quantify pluteus morphology, spicule length and spicule:body length ratio (Supplementary Table S1).

**FIGURE 4:**
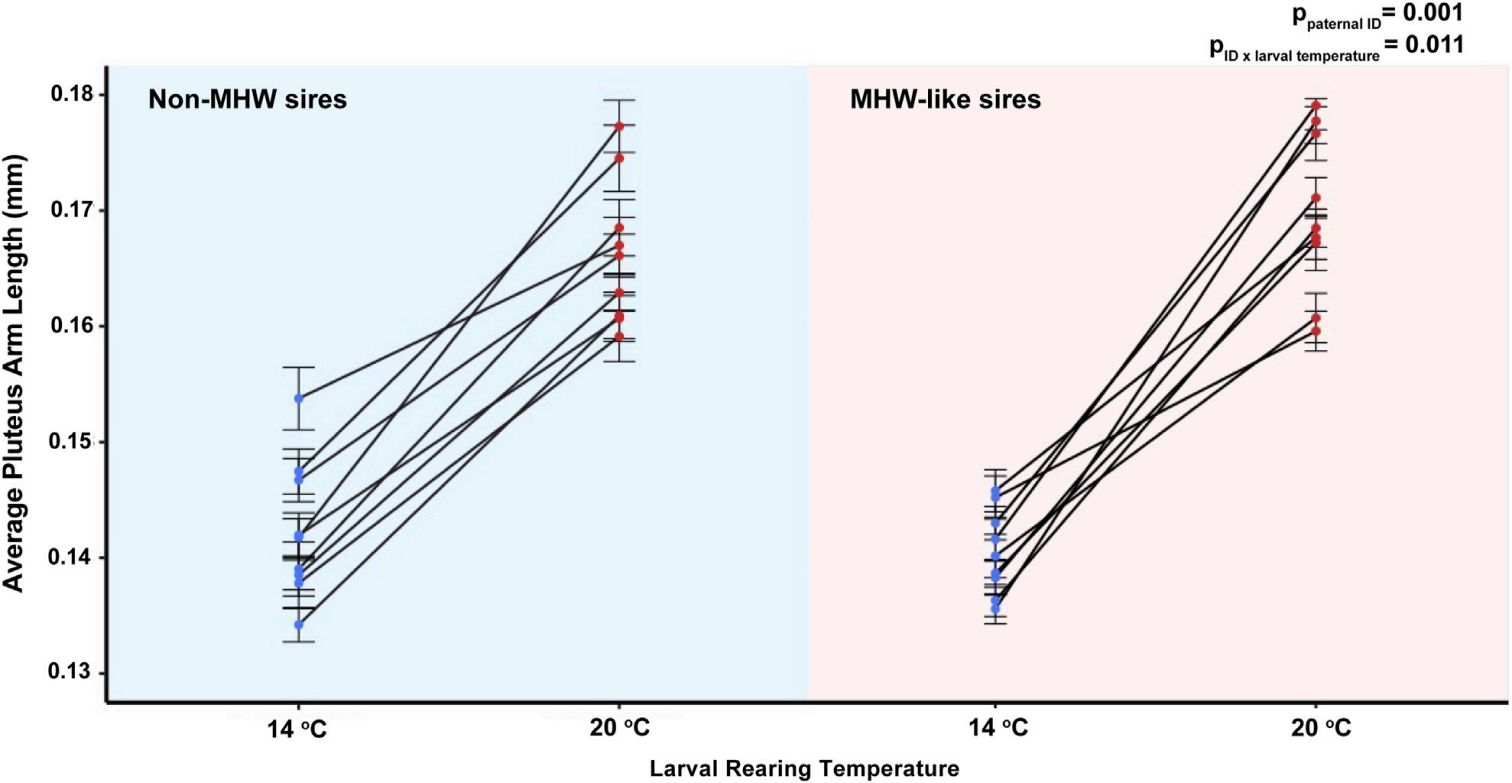
The effect of individual male identity and larval temperature treatment (14°C or 20°C) on echinopluteus arm length in the purple sea urchin, *Strongylocentrotus purpuratus*. Datapoints represent average arm lengths of offspring produced by individual sires (n = 9 individual male urchins/paternal treatment) and plots are separated by paternal acclimation temperatures, non-MHW (14°C) and MHW-like (20°C). Error bars are representative of mean ± standard error. ANOVA results with significant *p-*values are displayed on the graph.

Despite the minimal effect of paternal thermal experience on the size of larval offspring, there was a notable effect as a function of individual males. Here, paternal identity (e.g., specific individuals) (F_1,16_ = 3.52, *p* = 0.001) as well as the interactive effect between paternal identity and larval rearing temperature, significantly influenced larval size (F_1,16_ = 2.528, *p* = 0.011). Larvae from individual males varied in arm length across developmental and paternal temperature treatments (Figure 4). Of particular note was the decreased amount of variability in average arm length between offspring sired by MHW-like temperature males but raised under non-MHW temperature conditions (Figure 4).

## 4 Discussion

In this study of paternal effects under the elevated temperatures associated with MHW events, two clear findings emerged: 1) *S. purpuratus* larvae were most thermally tolerant when development occurred at a temperature that matched that of the paternal acclimation; and 2) larval size was not influenced by paternal thermal history but was instead overwhelmingly affected by larval rearing temperature, with higher temperatures producing significantly larger offspring. Below we discuss each aspect of the study and conclude by framing the results in the context of present-day MHW events in the Santa Barbara Channel and those predicted into the future.

### 4.1 Influences on larval thermal tolerance: paternal acclimation and larval rearing temperature

In this study, we observed one of the first documented cases of an adaptive paternal effect in sea urchins, where thermal tolerance of the larvae was increased when the thermal environment during development matched that of their sire’s acclimation temperature. Specifically, *S. purpuratus* larvae raised at an elevated temperature, 20°C, possessed significantly higher thermal tolerances when their sire was acclimated to 20°C as opposed to 14°C. This trend was reversed for larvae raised at non-MHW conditions of 14°C, where larvae whose sire was acclimated to 14°C exhibited a slight increase in thermal tolerance in comparison to larvae from 20°C-acclimated sires. This pattern appeared to be largely driven by the larvae of MHW-like temperature males, which exhibited a ∼1°C increase in LT_50_ values (the temperature in which 50% of larval mortality was observed) when reared at 20°C compared to 14°C. Alternatively, males acclimated under non-MHW conditions sired offspring that demonstrated minimal difference in LT_50_ values regardless of rearing temperature.

These results on early-stage *S. purpuratus* join a rich literature in larval ecology and the role of parental effects in the ecological development of marine organisms (Byrne et al., 2008; Ross et al., 2016; Parker et al., 2017; Donelson et al., 2018; Gibbs et al., 2021 - larval responses reviewed in Byrne, 2011). The broader literature indicates that parental effects, in general, can influence offspring success both positively or negatively, but the extent of these effects depends on the nature of stressor itself (e.g., duration, intensity, variability) as well as the contributions made by both dams and sires (Donelson et al., 2018; Yin et al., 2019). Our results add to a small, but growing body of evidence that the paternal environment is relevant and thus perhaps merits additional research in climate change contexts (Crean and Bonduriansky, 2014; Marshall, 2015).

Adaptive paternal effects have also been observed in marine fish and invertebrate taxa under varying environmental conditions including competition, salinity, and temperature (Crean et al., 2013; Jensen et al., 2014; Yin et al., 2019; Chang et al., 2021). In general, one of our results, that of increased thermal tolerance when offspring and paternal temperature match, supports the theory behind anticipatory maternal effects, where positive benefits to offspring are passed from their mother if their parent can predict what environment their offspring will encounter (Marshall and Uller, 2007). This scenario posits a cost for inaccurate predictions from the parent, which was witnessed in this study addressing paternal effects. In our analysis, the lowest LT_50_ values observed in this study across all treatments were in larvae that were sired by 20°C -acclimated males but developed at 14°C. Mechanistically, understanding how the males were possibly predicting the physical environment is outside the scope of this project. One aspect of this experiment to note is that the male urchins were removed from all abiotic *in situ* cues and experienced static temperatures during the laboratory acclimation, not reflecting the natural variability they likely encounter in nature. As such, the ability of our males to “predict” what their offspring might face could be driven by this month of unchanging thermal conditions, although there is much to be learned about what timescales and fluctuations influence these predictive effects.

Regardless of parental experience, pluteus larvae in this study had higher thermal tolerance when they were reared under elevated temperatures (Figure 2). This is likely due to the activation of the defenosome in the larvae, with genes of the general stress response being activated (Sconzo et al., 1995; Runcie et al., 2012; Shi et al., 2020). This priming of the urchin larvae’s heat response pathway earlier in development could prove beneficial for the persistence of latter developmental stages. In the Mediterranean mussel, *Mytilus galloprovincialis*, though, decreased expression of the heat shock gene, hsp90, in heat-acclimated sperm was correlated with increased developmental success in larvae raised under ambient conditions, but appeared to have a maladaptive effect on development for the same larvae under elevated temperatures (Lymbery et al., 2020; Lymbery et al., 2021). Such effects could be due to increased apoptosis, or cell death, triggered by prolonged synthesis of heat shock proteins (Takayama et al., 2003). Further exploration of the presence of hsp70 or other molecular chaperones within larvae from this study could shed more light into the mechanisms behind both the inter- and intragenerational plasticity in thermal tolerance observed here. Additionally, because sperm cells can serve as another source of RNAs and proteins, research into sperm’s role in priming larval thermal tolerance may prove of interest (Immler, 2018).

### 4.2 Effects of developmental and adult acclimation temperatures on larval size

Across marine invertebrate taxa, the temperature experienced during development is a dominant variable determining the size of embryos and larvae (Marshall et al., 2012). In this study, larvae that developed at higher temperatures (+7°C) were significantly larger than those in the non-MHW temperature treatment. This result is consistent with studies from various marine invertebrates, including other echinoderm, arthropod, and mollusk species (reviewed in (Byrne, 2011). For sea urchins, specifically, longer larval arms under higher temperatures appear in species found in tropical, temperate, and polar regions (Sheppard Brennand et al., 2010; Byrne et al., 2013; Wong and Hofmann, 2020). This trend may be stage-specific in urchins though, as similar temperature increases (+4°C) did not elicit any influence on the size of early developmental stages in the red sea urchin, *Mesocentrotus fransicanus*, until they became larvae (Wong and Hofmann, 2020).

The larger body sizes conferred by elevated temperatures can have a myriad of positive impacts on an individual’s survival and performance during early development. During the planktonic portion of many marine invertebrate life cycles, smaller individuals often take longer to settle and are more likely to be predated upon (Allen et al., 2008; Byrne et al., 2009; Ross et al., 2011). Here, we measured the length of early feeding arms in echinopluteus larvae, with those reared at 20°C possessing longer arms than those at 14°C. In echinoderm larvae, ciliated arms provide an effective means to capture algae for consumption. This increased surface area associated with longer arms translates to increased feeding success (Chan et al., 2011). The longer arms and faster development of larvae raised at 20°C—these individuals reached the echinopluteus stage ∼12 h sooner than those at 14°C - in this study highlight potential advantages of larger body sizes for larvae developing under elevated temperature scenarios (i.e., less time before settlement, or increased energy reserves).

Parental environment can also be an important driver of offspring size, but there is little evidence for a role of paternal environment alone. This lack of evidence stems generally from an absence of studies exploring the sole impact of paternal environment on offspring phenotype in marine invertebrates. At this time, the only exception is in the marine polychaete, *Hydroides diamphus*, where males exposed to more acidic conditions sired offspring with reduced juvenile growth rates (Lane et al., 2015). Paternal effect studies in marine invertebrate taxa have instead focused on how paternal environment influences other elements of offspring success such as fertilization success, larval survival, and developmental abnormality (Crean et al., 2013; Jensen et al., 2014; Guillaume et al., 2016; Leach et al., 2021). More often, marine invertebrate studies do not attempt to disentangle the distinct impacts of paternal and maternal environment on offspring phenotype. Such experiments have shown that general parental exposure to various environmental conditions positively influence offspring size and growth rates (Parker et al., 2012; Minuti et al., 2021). In purple sea urchins, female acclimation to varying temperature x pH regimes had no influence on echinopluteus larvae size, despite variation in egg lipid content (Wong et al., 2019; Strader et al., 2020). Echinopluteus arm length may therefore be a metric that is more heavily influenced by the offspring’s current environment as opposed to a carry-over effect from either parents’ past experiences.

## 5 Conclusion

The results of this laboratory study demonstrated that the temperature experienced by male urchins during gametogenesis can influence the performance of their progeny. Furthermore, this effect appeared after only a 28-day acclimation period, an exposure period that matches recorded MHW events in the SBC (Cavanaugh et al., 2019; Chamorro et al., 2023). While MHW temperatures alone could serve to elicit tolerance in *S. purpuratus* early developmental stages, the overlapping phenology of MHW events and gametogenesis of purple urchins may amplify the mismatches between paternal and offspring environment. For example, temperatures of >20°C during an early fall MHW event (and the start of gametogenesis for sea urchins) may be followed by temperatures closer to 13°C in the winter, when larvae would be in the water column. From the results shown here, thermal mismatches of this magnitude may prove detrimental, depressing performance of future populations (but see Minuti et al., 2021).

Overall, observations made in this study indicate that the interaction between the timing of MHWs and life history events will be key in forecasting the outcome of community structure changes within coastal marine ecosystems such as kelp forests. In general, the response of urchin population and recruitment as it relates to environmental temperature has been show to vary between geographic locations across California (Okamoto et al., 2020). In our region, urchin recruitment in the SBC was negatively correlated with sea surface temperatures, results that are in line with decreases in *S. purpuratus* population size following the 2014–2016 MHW event mentioned above (Reed et al., 2016). In northern California, the combination of MHWs and substantial increases in the local *S. purpuratus* population underpinned a massive shift in ecosystem state from dense kelp forests to sea urchin barrens (Rogers-Bennett and Catton, 2019). This variance in the MHW response of Californian *S. purpuratus* populations may stem from other factors than the temperatures larvae experienced themselves, included parental environmental history. In addition, the complexity of the physical environment, e.g., the interaction of upwelling and MHWs (Rühmkorff et al., 2023) and bottom MHWs at sites such as kelp forests (Amaya et al., 2023), underscore that MHWs will be a force structuring coastal marine ecosystems in the future.

From a physiological perspective, research into the gametes of acclimated adults represents an interesting place to start for thinking about mechanisms driving potential adaptive parental effects. Studies determining the degree of gamete plasticity elicited by different adult acclimation conditions as well as how differences within gametes influence later development could shed light onto this process. For sperm specifically, there is exciting evidence to suggest that these cells may harbor traces of the paternal environment through epigenetic marks such as DNA methylation that may influence such marks in developing offspring (Jiang et al., 2013; Immler, 2018). Additionally, sperm plasticity may elicit adaptive effects via the activity and motility of sperm cells themselves within their environment (Chirgwin et al., 2020; Abdelgalil et al., 2022). Given the influence of temperature and other environmental factors on fertilization, both itself and via parental conditioning, diving deeper into sperm morphology and performance under extreme event scenarios could prove a rich area of future research (Sewell and Young, 1999; Leuchtenberger et al., 2022).

## Data Availability

The datasets presented in this study can be found in online repositories. The names of the repository/repositories and accession number(s) can be found below: https://github.com/tleach32/Spurp-MHW-Larval_2023.git.
