# Supplementary material for: Marine heatwave temperatures enhance larval performance but are meditated by paternal thermal history and inter-individual differences in the purple sea urchin, Strongylocentrotus purpuratus
Source: Front Physiol. 2023 Aug 4;14:1230590. doi: 10.3389/fphys.2023.1230590 (PMC10436589; doi:10.3389/fphys.2023.1230590)
Supplement: Supplementary file 1 [file Table1.docx]

Supplementary Material

Marine heatwave temperatures enhance larval performance but are meditated by paternal thermal history and inter-individual differences in the purple sea urchin, *Strongylocentrotus purpuratus*

## Supplementary Tables

**Supplementary Table 1.** Body morphometrics for *S. purpuratus* echinopluteus larvae. All values given as mean ± standard deviation.

| Adult Temperature Treatment | Developmental Temperature Treatment | Pluteus Spicule Length (mm) | Pluteus Arm Length (mm) | Spicule:Body Length Ratio |
| --- | --- | --- | --- | --- |
| non-MHW (14 ^o^C) | non-MHW (14 ^o^C) | 0.142 ± 0.020 | 0.142 ± 0.014 | 0.998 ± 0.063 |
|  | MHW-like (20 ^o^C) | 0.179 ± 0.021 | 0.166 ± 0.018 | 1.078 ± 0.058 |
| MHW-like (20 ^o^C) | non-MHW (14 ^o^C) | 0.142 ± 0.018 | 0.141 ± 0.012 | 1.004 ± 0.062 |
|  | MHW-like (20 ^o^C) | 0.181 ± 0.018 | 0.170 ± 0.017 | 1.063 ± 0.045 |
